# Judgment an Important Factor in the Preservation of Teeth

**Published:** 1895-11

**Authors:** Frederick Bradley

**Affiliations:** Newport, R. I.


### JUDGMENT AN IMPORTANT FACTOR IN THE PRESERVATION OF TEETH.[1]

BY FREDERICK BRADLEY, D.M.D., NEWPORT, R. I.

THE world is greatly indebted to the enthusiasts, extremists, and radicals, and, paradoxical as it may seem, the world is also greatly indebted to men of moderate views, the judicial and the conservative.

A lady from abroad, who is prominent in public affairs, recently said that when a man stands alone for his belief or ideas he is called

---

[1] Read before the American Academy of Dental Science, Boston, March 6, 1895.

a fanatic; when a limited number are his disciples, he is called an enthusiast; but if his belief or ideas are worthy of general acceptance, he is a hero.

Abundant proof can be furnished showing this to be true in all professions, particularly in theology and medicine. Hahnemann, who may have been looked upon as a fanatic or an enthusiast, is now held to be something of a hero by at least a part of every community, and it is generally conceded, I think, that his influence on general medicine has been, on the whole, beneficial.

While it may be said, broadly speaking, that all great reforms, and even innovations, need the enthusiasm and earnestness of the fanatic to arrest the attention of the masses, it is equally true that not every instance of fanatical, or even enthusiastic, devotion to an idea is proved in the end to be founded on true merit; and it is an excellent safeguard which the average mind of the masses interposes between the chimerical and the welfare of humanity, refusing to follow or to be led by every will-o'-the-wisp who labors under the impression that he has a mission to fill.

No doubt many of the isms which come to the front at one time or another contain the germ of some idea valuable in itself and of undoubted service when used with judgment. But, on the contrary, if it should be taken up by the extremist or radical, its real worth is frequently lost entirely, or so obscured by exaggerated claims that its period of usefulness is much curtailed.

Before applying these principles to our own profession let us look a few moments at the material which we have to work upon and the end which we hope to accomplish. In my student days I remember hearing a prominent professor make the following statement concerning the human eye: "If the modern oculist was to make as imperfect an optical instrument as the average human eye, the probabilities are his occupation would be gone; but," said he, "we must bear in mind the nature of the material from which the eye is made, how it is subject to the laws of decay and repair, and, taking all things into consideration, it is undoubtedly a good organ."

If this is true of the eye, what may be said respecting the teeth? The influence of one's ancestors, according to the laws of heredity, the large or small number of children in a family, well or poorly nourished condition, the influences of a varying diet, the occupation of the individuals, sedentary or otherwise, the care exercised in cleansing, the ever-varying condition of the saliva,—all these conditions are important factors in determining our method of procedure.

And what do we hope to accomplish? First, the relief and prevention of pain; secondly, the preservation or restoration of useful organs of mastication; thirdly, to retain or develop an attractive appearance of the mouth.

Bearing these conditions in our mind, is it not presumptuous of any dentist to claim the ability by any single special or individual method to accomplish the desired end? And yet we do hear of men becoming so wedded to an idea or a method of work that their enthusiasm leads them to ignore, and sometimes to decry, much that is good and useful, because, forsooth, it runs counter to their pet theory.

Let me go a little more into details. Is it wise for a dentist to boast that under no circumstances would he extract a tooth or advise its removal? I may be mistaken, but I do believe we may in many cases do our patients the greater service by a judicious use of the forceps. It may be in a case of regulating, or in a persistently troublesome case of pyorrhœa alveolaris, or, possibly, simply to relieve pain in stubborn cases, or in the mouths of those unable to pay for more conservative treatment, and yet we hear occasionally of some one who claims he can save all the teeth, and it is the duty of all dentists to do so. Twenty-eight teeth, or even less, in fairly good condition are preferable to thirty-two crowded and ill-conditioned teeth.

It is my privilege as an instructor to encourage students to ask questions and think for themselves. I have been asked such a question as the following: "What is the best way to treat and fill a pulpless tooth?" "Because," said the student, "I want to know the best way, and I will do it so every time." My reply is, I know of no way that is best in all cases; that as the "punishment must fit the crime," so the remedy should be according to the diseased condition, bearing in mind the desired result,—viz., a perfectly aseptic condition of the pulp-chamber and canals and a filling in the root that will exclude moisture, and I think an experienced judgment will not always suggest the same methods or material.

A few years ago there was quite a hue-and-cry about implantation. Many demonstrations were given, papers were read describing and advocating the operation. Some of the more enthusiastic advanced such exaggerated claims for this operation that, compared with the results shown later, such claims were, to say the least, unwise. I believe there is something of value in it,—there are cases where the successful implantation of a tooth would be a consummation to be most ardently desired,—but to recommend the implantation of a few roots and the attachment thereon of extensive pieces of bridge-work certainly seems to be the dream of the fanatic.

And this brings us to the consideration of bridge-work from the stand-point of this paper. Do we not see evidences of the presence of the fanatic frequently in our own practice, and daily in the advertisements of the daily papers? That the insertion of suitable pieces of bridge-work is a legitimate undertaking, both movable and immovable, no doubt we should all agree. But should we not hesitate and make haste slowly when it comes to cutting off and grinding down good and useful teeth for the purpose of inserting what may be merely an experiment? Have not the extravagant claims of these fanatics in a measure disgusted, or at least discouraged, the more conservative, causing them to hesitate in attempting anything in this line? Here is a field for the exercise of an experienced judgment, that will carefully weigh the advantages and disadvantages impartially, and that does not look upon a difficult and complicated case as an opportunity either for experimentation or to show off the skill of the gold-worker.

Possibly in the use or choice of filling-materials and the methods of manipulation do we find the most important occasions, when we should use judgment in our work. When we hear of men, prominent in the profession, who openly speak of their intention to confine themselves to the use of plastic filling-materials, and others who think no metallic filling should be made in the teeth until the patient is upward of twenty years of age, and, on the other hand, equally prominent and conscientious men who use gold and other metals even in deciduous teeth, certainly it is not surprising that the young student should ask for some authoritative instruction as to the course he should pursue. For my own part, I believe we should be guided by the conditions presented. The indiscriminate use of gold in teeth of a poor texture, whether for children or for persons in delicate health, must undoubtedly cause much suffering, and very frequently fail to accomplish the purpose in view; and, on the other hand, to continue the use of plastic materials when the teeth are hard and of good texture seems an unnecessary waste of time, as the work will need frequent replacing.

Let me instance a case. Suppose the tooth to be of a fair quality, the patient twelve years old, the deciduous teeth gone, the six-year molars filled on grinding-surface with cement, and we find small or medium-sized cavities on the mesial surface. In such a case, would it not be better to make a gold filling on the mesial surface, because of its exposed view, and leave the cement filling on the grinding-surface till it needs further attention ?

As to methods of manipulating gold, we are told by one that purely soft gold, rolled into cylinders and wedged into the cavity, is the best; another will use cohesive foil entirely, with automatic engine or electric mallet; another uses no mallet, but thinks hard pressure is the ideal method; and only recently we listened to a most interesting paper claiming that gold should be burnished into the cavity. In answer to the student who asks which is the best method, what shall we say? I think it well to advise him to become expert in each method of manipulation; and the probabilities are that nine out of ten students will not use any one method exclusively when they have been in practice five years, but a combination of two or more methods, because their experience has taught them how to work to the best advantage in saving their own strength with the minimum of discomfort to the patient.

Some time ago a dentist told me that he had not only discontinued the use of carbolic acid and creosote, but he had cleared them out of his office. Possibly these drugs may have been superseded by some others for certain purposes, yet I venture to say there are occasions where one or the other might be used to advantage. In the summer months I occasionally see a lady whose teeth are said to be in the care of a prominent Boston dentist. More than once the trouble has been with pulpless teeth, and she states, with every confidence, that unless the canals are filled with cotton and creosote she knows they will be troublesome. Now, while I may not agree with the idea that cotton and creosote are the best to use for a root-filling, I allow the lady to have her own way, and she leaves my office free from pain and satisfied in her mind.

And right here I wish to say a few words as to accepting the suggestions of the patient. No doubt we all have patients who feel called upon to suggest little things at times, and I think we have a duty to perform in deciding aright how far we shall listen to such. Of course, if we are consulted, we very naturally expect our advice to be followed, and wherever a principle is involved we must decide for ourselves and the patient; yet there are occasions when it is well to heed the expressed preferences of the patient. I have known of a case where a young person, needing to have a tooth removed with the use of an anæsthetic, preferred cocaine to be injected; but his mother wished him to have nitrous oxide. After the operation nothing could convince the boy but he knew all the details of the operation, and I have no doubt the result would have been more satisfying to him had we used cocaine. While it should be our intention to do all our work so thoroughly that it may stand the most rigid examination, there are occasions when our judgment should indicate that we must be satisfied with doing the best we can under the circumstances, bearing in mind that all patients cannot, or will not, submit equally well to the necessary pain and fatigue generally experienced in building up show-pieces of our work.

Some time ago I was very frequently in the company of a number of young school-teachers, and I was impressed very forcibly with the idea that some of them seemed to think—or, at least, act and talk as if they thought—that the scholars in a school were sent for the purpose of supplying raw material whereon the teacher might develop or exhibit some special theory of teaching, instead of being there to acquire an education. I think we should never use our patients for such a purpose. Let us make beautiful gold fillings where suitable, make and insert crowns and bridge-work, and, when a cultivated judgment indicates it, use the plastic material for fillings. Do not be afraid to say that in our judgment such or such a course would be the best to pursue.

I know sometimes the young school-teacher would be greatly annoyed because a pupil less quick to apprehend the instruction than the average of her class interfered somewhat with the development of the pet theories; and if we treat all our patients in the same inflexible way, we shall find that our pet theories are sometime completely overthrown.

It cannot be supposed for a moment that the various methods of operating or manipulating are the result of accident, any more than the different filling-materials came by chance, or the remedies used in our Pharmacopœia are the effects of a blunder. Much has been developed because the spur of necessity acted as an almost unfailing stimulus to progress; so let us not throw the results to one side hastily, for there is generally something of value in all processes and remedies.

To recapitulate, the usefulness of the enthusiast or radical consists largely in calling the attention of the general public to what is new, or a new use of something old. The usefulness of the moderate or conservative man is to weed out the good from the bad, and, in a measure, act as an antidote to the enthusiast.

The injury which the enthusiast may do consists in over-statement and extravagant claims, thereby leading the unwary and easily influenced astray. The injury which the conservative may do is in resisting the new because it is new, and in thinking and saying that the old ways are good enough for him.

So, in our profession, those who cultivate a fad or ride a hobby, whether it be the ignorant charlatan who wields the forceps with more zeal than discretion for the purpose of making an artificial substitute, or his opposite, who never extracts a tooth; the boastful bridge-worker, who will make a full set of teeth and fasten them to four or five roots, or the man who never attempts even to put on a crown; the renowned gold-worker, who believes in the precious metal first, last, and all the time, or his equally renowned professional brother, who never uses gold, but believes in gutta-percha and the cements,—all these should, we believe, make the exercise of judgment a prominent factor, modifying arbitrary methods and their use of materials in a cast-iron rule.

